# Nomogram for Predicting Long-Term Survival after Synchronous Resection for Hepatocellular Carcinoma and Inferior Vena Cava Tumor Thrombosis: A Multicenter Retrospective Study

**DOI:** 10.1155/2020/3264079

**Published:** 2020-04-08

**Authors:** Yannan Bai, Jiayi Wu, Yong Zeng, Jie Chen, Shuangjia Wang, Shi Chen, Funan Qiu, Songqiang Zhou, Shen You, Yifeng Tian, Yaodong Wang, Maolin Yan

**Affiliations:** ^1^Department of Hepatobiliary Surgery, Fujian Provincial Hospital, Shengli Clinical Medical College of Fujian Medical University, Fuzhou 350001, China; ^2^Department of Hepatic Surgery, West China Hospital, Sichuan University, Chengdu 610041, China; ^3^Department of Hepatic Surgery, Sun Yet-sen Memorial Hospital, Sun Yet-sen University, Guangzhou 510120, China; ^4^Department of Hepatobiliary & Pancreatovascular Surgery, First Affiliated Hospital of Xiamen University, Xiamen 361003, China

## Abstract

**Background:**

Although surgery for hepatocellular carcinoma (HCC) complicated with inferior vena cava tumor thrombus (IVCTT) may improve survival for some patients, prognostic markers remain elusive because of its rarity. We constructed a prognostic nomogram which predicts individualized survival benefit of curative-intent surgery for HCC patients with IVCTT.

**Methods:**

According to abdominothoracic anatomy of inferior vena cava (IVC), IVCTT can be divided into 3 types: inferior diaphragmic (ID), superior diaphragmic (SD), and intracardiac type (IC). Data of 64 HCC patients with IVCTT who underwent curative-intent surgery between 2008 and 2015 in four centers in China were analyzed retrospectively. Univariate and multivariate Cox regression analyses were conducted to select variables for the construction of a prognostic nomogram. Predictive accuracy and discriminative ability were examined by concordance index (C-index) and calibration curve.

**Results:**

Of 64 patients in the IVCTT classification, 37 (57.8%) were classified as ID type, 15 (23.4%) as SD type, and 12 (18.8%) as IC type. The 1-, 2-, 3-, and 5-year disease-specific survival (DSS) rates for patients in ID, SD, and IC groups were 94.4%, 55.6%, 71.4%, and 30.0%; 27.8%, 21.4%, 7.1%, and 0%; and 8.3%, 0%, 0%, and 0%, respectively. Independent factors included in the nomogram were ECOG performance status, AFP level ≥ 400 *μ*g/L, tumor size ≥ 10 cm, portal vein tumor thrombosis, and IVCTT classification. The C-index of the nomogram was 0.812 (95% CI 0.761–0.873). The calibration plot for DSS probability showed excellent agreement between the prediction by nomogram and actual observation.

**Conclusions:**

Curative-intent surgery should be carefully evaluated and suggested according to our novel IVCTT classification. We have developed a visual web-based nomogram model to predict oncological prognosis of curative-intent surgery for HCC patients with IVCTT.

## 1. Introduction

In most circumstances, inferior vena cava tumor thrombus (IVCTT) originates directly from a tumor thrombus in hepatic veins (HVs) and extends in a manner similar to that established for hepatic vein tumor vein (HVTT) in hepatocellular carcinoma (HCC) [1–5]. It is classified as an extremely advanced disease; the prognosis is dismal with a reported median survival time (MST) of 2 to 4 months in the absence of treatment [2, 5–11]. HCC patients with IVCTT are faced with a series of unfavorable complications, including sudden death due to heart failure or pulmonary embolism caused by a dislodged thrombus [12, 13], as well as extrahepatic metastases due to direct cancer cell dissemination into the systemic circulation [14]. According to current HCC guidelines, palliative or systemic therapy is the proposed therapeutic strategy for HCC patients with IVCTT [15–18].

Surgery is rarely suggested because of the advanced stage of IVCTT. Curative-intent surgery is technically demanding and carries high surgical risk. Recently, several series of HCC patients with IVCTT were documented with promising prognostic outcomes when they received aggressive surgery, with an MST ranging from 18.1 to 30.8 months [3–5, 9, 19–21]. In selected settings, patients might even gain long-term survival benefits from curative surgery such as in case of patients with good liver function reservoir, resectable primary tumor and vascular thrombus, and inexistence of extrahepatic metastases [4, 20]. Therefore, surgical resection of HCC with IVCTT should be considered as curative treatment option. However, invasiveness of curative-intent surgery for HCC patients with IVCTT may vary a lot according to the location of the intrahepatic tumor and, more importantly, the extent and length of the IVCTT. Apart from several surgical methods documented in case reports [22–24], the most useful references for IVCTT classification are the systems proposed by Li et al. [4] and Sakamoto and Nagano [21]. This classification system makes surgical decision-making simple, but it fails to covering the whole distribution of tumor thrombus in the IVC.

Here, we present a novel classification system for IVCTT, based on the source of the tumor thrombus and the abdominothoracic anatomy of IVC between the renal veins and the right atrial entrance. We subsequently reviewed 64 HCC patients with IVCTT who underwent surgical resection at 4 tertiary hospitals, taking note of IVCTT classification, surgical perspectives, and prognostic outcomes. The purpose of this study was to develop a visual web-based nomogram model to predict oncological prognosis of curative-intent surgery for HCC patients with IVCTT.

## 2. Methods

### 2.1. Patients

We performed a retrospective review of 64 patients, who underwent synchronous resection of primary hepatic tumor and IVCTT at the Fujian Provincial Hospital (*n* = 11), West China Hospital (*n* = 27), Sun Yet-sen Memorial Hospital (*n* = 21), and First Affiliated Hospital of Xiamen University (*n* = 5) between January 2008 and December 2015. HCC was diagnosed according to the practice guidelines recommended by the American Association for the Study of Liver Diseases [11]. The diagnosis of IVCTT was established based on radiological or intraoperative findings, further confirmed by pathologic examination (Figure S1). Due to the absence of the standard therapeutic modalities for HCC with IVCTT, any adopted treatment was performed under the formal agreement of patients and their relatives after being thoroughly informed of the detailed pros and cons of surgery and nonsurgical treatments. In this study, surgery was selected for each patient based on curative intention, with no requirement of additional ethics approvals, and data were evaluated anonymously under permission from the included hospitals.

### 2.2. Definitions and Classification

**Figure 1:**
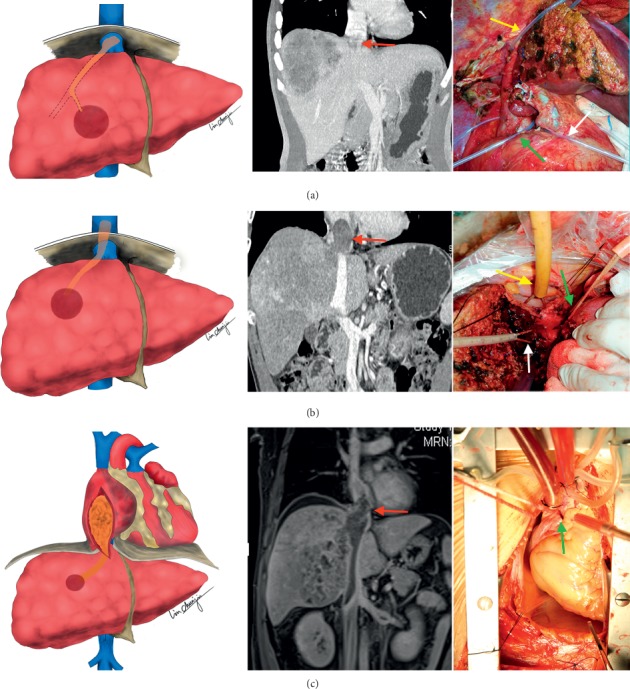
The diagram of the IVCTT classification presented with its diagnostic image (middle panel) and preconditioning of hepatic vasculature and the IVC intraoperatively (right panel). (a) Inferior diaphragmic type (type ID): the extent of IVCTT is below the level of diaphragm; taping of hepatoduodenal ligament (white arrow), infrahepatic IVC (green arrow), and suprahepatic IVC (yellow arrow); (b) superior diaphragmic type (type SD): the extent is above the level of diaphragm but still outside the right atrium; taping of hepatic veins (diseased side, white arrow; contralateral side, green arrow) and supradiaphragmatic IVC (yellow arrow); (c) intracardiac type (type IC): the body of IVCTT extends into the right atrium; atriotomy cardiopulmonary bypass by cannulation of the ascending aorta and superior vena cava (green arrow).

In anatomy, the abdominothoracic inferior vena cava (IVC) can be divided into several sections according to anatomic landmarks, including the renal veins, accessory HVs or short HVs, the main HVs, the diaphragm, and the entrance of the right atrium (RA). Accordingly, there is a natural division into 3 types defined by the location and extent of IVCTT. The details of our classification system include (1) *inferior diaphragmic type* (type ID), where the extent of IVCTT is below the diaphragm, (2) *superior diaphragmic type* (type SD), where the extent is above the level of the diaphragm but still outside of the RA, and (3) *intracardiac type* (type IC), where the body of the IVCTT extends into RA (Figure 1).

### 2.3. Surgical Procedure

Synchronous resection of primary tumor and IVCTT was carried out via a right subcostal incision with a midline extension combined with xiphoid extension (if necessary) [4, 5]. The liver was mobilized completely by division of the bilateral ligamentous attachments, facilitating exploration and later operation of total hepatic vascular exclusion (THVE). Intraoperative ultrasonography as well as transesophageal ultrasonography (in most cases) was routinely performed for the detection of intrahepatic metastases, the location and extent of tumor thrombus in liver-related macrovasculature, and the demarcation of the parenchymal transection plane. After detective manipulation, hepatoduodenal ligament and the infrahepatic and suprahepatic IVC were encircled with umbilical tapes in preparation for THVE. The taping site of the suprahepatic IVC should take into consideration the cranial extent of the IVCTT, preventing the thrombus from dropping down or rupturing into the blood circulation. Hepatectomy was performed under selected occlusion of the affected side of hepatic blood inflow or total hepatic inflow occlusion (Pringle's maneuver), leaving only the hepatic vein that had filled with tumor thrombus connecting the liver mass and IVC on either side. Precise division of hepatic parenchyma between the dissected plane and the IVC was required to provide enough space for a longitudinal opening of the IVC and hepatic vein under direct visualization. Thrombectomy of IVCTT was then performed under well-controlled THVE.

### 2.4. Technical Features

Properly positioned taping of the hepatic vasculature and the IVC for subsequent THVE is a critical step for *en bloc* thrombus resection. In most circumstances, encircling and taping of the hepatoduodenal ligament and infrahepatic IVC are established at each respective site using a similar procedure [5, 22]. However, the taping site of the cranial IVC is variable, depending on the cranial location of the IVCTT (Figure 1, right panel). For type ID, the occlusion of the cranial IVC is located at the suprahepatic IVC, just under the diaphragm, since the cranial extent of the thrombus is limited to a section of the inferior diaphragmic IVC (Figure 1(a)). For type SD, the occlusion of cranial IVC should be moved up to the intrathoracic IVC section, via combined thoracoabdominal incision at the pericardium and diaphragm with various dissecting approaches, as documented previously (Figure 1(b)) [24]. These approaches exempt patients from the massive injury of a median sternotomy, yet they provide satisfactory exposure of the intrathoracic IVC. For type IC, the cranial extent of the thrombus extends into the RA. Thus, an atriotomy is required under the establishment of a cardiopulmonary bypass (Figure 1(c)).

### 2.5. Statistical Analysis

The primary outcome of interest was disease-specific survival (DSS) in relation to several potential risk factors. The baseline characteristics of patients are documented as medians with range. The Wilcoxon rank-sum test was used to compare continuous variables, and the Pearson chi-square test was used to compare categorical variables. Survival curves were described using the Kaplan–Meier method, with survival comparisons using the log-rank test. Univariate analyses were performed using Cox regression models, with statistically significant variables selected for inclusion in multivariate Cox regression modeling.

A visual web-based nomogram was created based on the significant variables in the multivariate analysis. A final model was selected by a backward step-down selection process based on the Akaike information criterion (AIC) [25]. The performance of the nomogram was measured by Harrell's C-index for model accuracy prediction, and calibration plots were generated showing the consistency of the model predictions with the actual observed events, using a bootstrap method with 1,000x resampling [26]. Statistical analyses were performed using R version 3.5.1 with the package of survival, survminer, rms, and DynNom (http://www.r-project.org/). Statistical significance was indicated by *P* < 0.05.

## 3. Results

### 3.1. Clinicopathologic Characteristics of Patients

The median age of patients at surgery was 53 (range: 31–78) years. According to IVCTT classification described above, the numbers of patients in the ID, SD, and IC groups were 37 (57.8%), 15 (23.4%), and 12 (18.8%), respectively. Most patients had good ECOG PS (0 score, 65.6%) and Child-Pugh grade (A grade, 87.5%). More than half of the patients (54.7%) had concomitant portal vein thrombosis (PVTT). In addition, major hepatectomy was performed in 40 patients (62.5%), with achievement of R0 resection in 27 patients (42.2%); all of them from IVCTT patients of ID and SD, while none of IC patients achieved R0 resection.

Baseline patient characteristics and clinical variables for the 3 types are listed in Table 1. There were no significant differences in most clinicopathological variables between ID and SD patients, with the exception of ECOG performance score (ID vs. SD, *P*=0.01), or between SD and IC patients, with the exception of HCC tumor differentiation scores from pathology (ID vs. SD, *P*=0.004; SD vs. IC, *P*=0.04).

### 3.2. Perioperative and Long-Term Oncological Outcomes after Surgical Treatment

A comparison of the perioperative and long-term oncological outcomes in the three groups is listed in Table 2. Median operative time for ID, SD, and IC patients was 260, 307.5, and 419 minutes, respectively, with significant differences between ID and SD groups (*P*=0.039) and SD and IC groups (*P*=0.004). There were no significant differences in other perioperative characteristics including blood loss, length of hospital stays, and morbidity rate.

In terms of long-term oncological outcomes, 49 patients (76.5%) died during follow-up period, 4 (6.25%) within 90 days postoperatively. The median DSS rates for ID, SD, and IC groups were 29, 14, and 8 months, respectively, with significant differences between ID and SD groups and between SD and IC groups (both *P* < 0.001).

**Figure 2:**
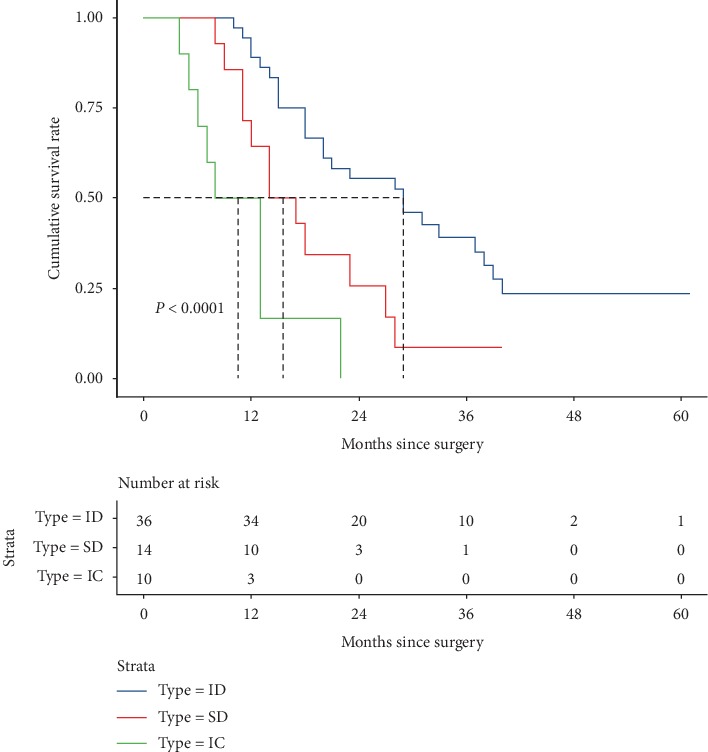
Kaplan–Meier overall survival plot for all HCC patients with IVCTT grouped by classified type.

The 1-, 2-, 3-, and 5-year DSS rates for patients in ID, SD, and IC groups were 94.4%, 71.4%, 55.6%, and 30.0%; 27.8%, 21.4%, 7.1%, and 0%; and 8.3%, 0%, 0%, and 0%, respectively (Figure 2 and Table 2). Compared with ID group, patients in SD and IC groups had decreased DSS after surgical treatment (HR 2.393, 95% CI 1.183 to 2.847, *P*=0.015 and HR 6.852, 95% CI 4.843 to 16.491, *P* < 0.001, respectively).

### 3.3. Multivariable Cox Regression Analysis of DSS

**Figure 3:**
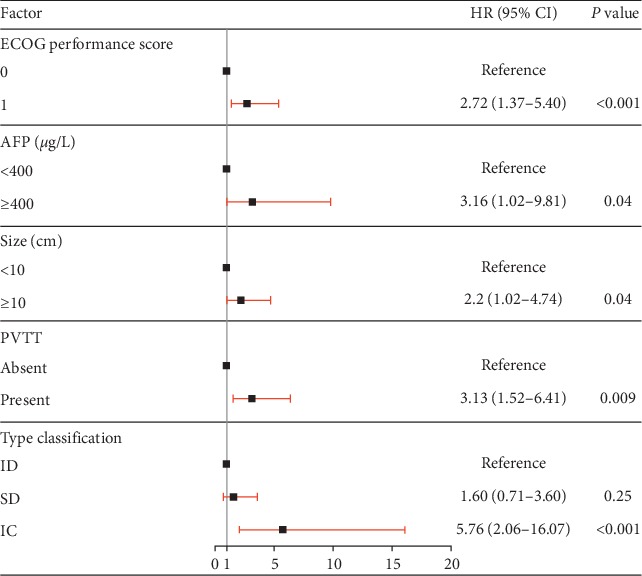
Forest plot of independent risk factors in multivariate Cox regression analysis.

The results of the univariable and multivariable analyses of DSS after surgical treatment for HCC patients with IVCTT are listed in Table 3. Significant variables (*P* < 0.050) in the univariable analysis were entered in the multivariable analysis which identified IVCTT classification as independent risk factors for reduced DSS (type SD, HR 1.60, 95% CI 0.71 to 3.60, *P*=0.25 and type IC, 5.76, 95% CI 2.06 to 16.07, *P*=0.001, respectively). Other independent risk factors for DSS were ECOG performance score (HR 2.72, 95 percent CI 1.35–5.40, *P*=0.004), AFP level ≥ 400 *μ*g/L (HR 3.16, 95 percent CI 1.02–9.81), tumor size ≥ 10 cm (HR 2.20, 95 percent CI 1.02–4.74), and PVTT status (HR 3.13, 95 percent CI 1.52–6.41, *P*=0.002) (Figure 3).

### 3.4. Prognostic Nomogram for DSS and Corresponding Visual Web-Based Model

**Figure 4:**
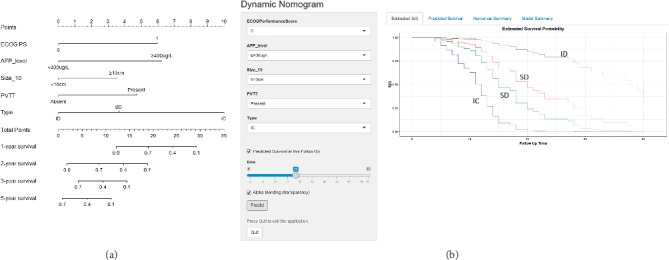
HCC patients with IVCTT survival nomograms. (a) Nomogram for predicting 1-, 2-, 3-, or 5-year survival probability. (b) Dynamic web-based nomogram model available at https://yannanbai.shinyapps.io/YanDSS/. To use this model, choose the value for each variable and the predicted survival time; then, press the “predict” button. PVTT: portal vein tumor thrombus.

**Figure 5:**
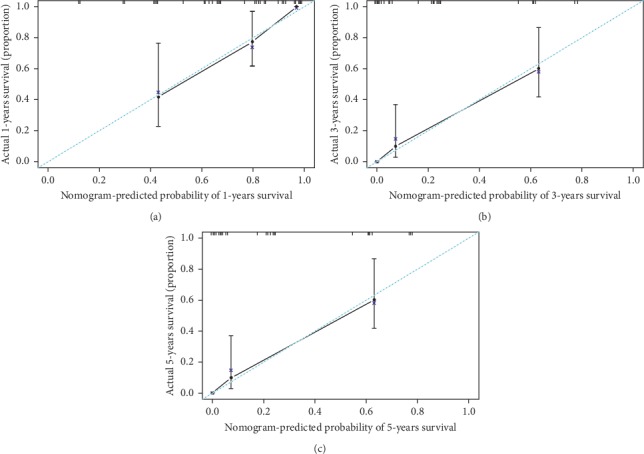
The calibration curve for predicting patient survival at (a) 1 year, (b) 3 years, and (c) 5 years. Nomogram-predicted probability of overall survival is plotted on the *x*-axis; actual overall survival is plotted on the *y*-axis.

The prognostic nomogram that integrated all significant independent factors for predicting DSS after surgical treatment for HCC patients complicated with IVCTT is listed in Figure 4(a). The C-index for the DSS prediction was 0.812 (95% CI 0.761–0.873). The calibration plot for the probability of survival at 1, 3, or 5 years after surgery showed an excellent agreement between the predictions by nomogram and the actual observation (Figure 5).

Since nomograms have been well developed for predicting prognosis in multiple cancers, a dynamic web-based nomogram was constructed to present survival probabilities for individualized HCC patients with IVCTT with substantial visualization (the YanDSS model, https://yannanbai.shinyapps.io/YanDSS/, Figure 4(b)).

## 4. Discussion

HCC complicated with IVCTT represents an extremely advanced disease due to macrovascular invasion which indicated poor survival; thus surgical treatments are not recommended by HCC guidelines. Furthermore, synchronous resection of primary liver tumor and IVCTT is technical demanding and will introduce high-grade surgical risks to patient. However, curative-intent surgery was positively tried in global tertiary liver centers with documented survival benefits ranging from MST 18.1 to 30.8 months, which is better than sorafenib or other systemic treatments [3–5, 9, 19, 20]. In theory, it is believed that only the surgery has the chance to completely remove primary tumor and IVCTT. Furthermore, the favorite long-term prognosis was achieved in parts of patients who had a resectable primary tumor and well-preserved physical performance status and liver function. However, long-term survival was not promised to all HCC patients with IVCTT, suggesting that more detailed clinical variables should be considered in defining surgical indication, especially tumor-specific characteristics.

Indeed, there are two similar tumor thrombus classifications concerning extent of IVCTT in HCC patients described recently [4, 21]. In detail, IVCTT was classified in 3 types of highly depending anatomy of the liver: type I, inferior (Li classification) or posterior (Sakamoto classification) hepatic type; type II, superior hepatic type; and type III, intracardiac type. Their classifications may develop confusion between types I (inferior or posterior hepatic) and II (superior hepatic). It is not precise enough in nomenclature because the extent of inferior or posterior hepatic type cannot cover the section of IVC between cranial surface of the liver and the diaphragm; conversely, the extent of superior hepatic type overlaps the section of IVC in type I mentioned above. Hence, we suggested that the nomenclature of IVCTT types I and II should be based on the anatomy of diaphragm (inferior diaphragmic, ID, and superior diaphragmic, SD). The diaphragm demarcates the cavity of the abdomen and the thorax. On surgical prospects, our classification is helpful in deciding operative approach, whether pure abdominal surgery (type ID, the extent of IVCTT below the diaphragm, Figure 1(a)) or combined thoracic surgery (type SD, the extent of IVCTT above diaphragm, Figure 1(b)). In our classification system, there are 37 patients with type ID, 15 patients with type SD, and 12 patients with type IC. And operative complexity can be indicated by operative time between ID, SD, and IC groups (Table 2).

The second important issue of this study was surgical outcomes analyses based on the IVCTT classification. In the present cohort, all the patients were treated with curative-intent surgery; however, complete resection of primary tumor and tumor thrombus is by no means an easy work, just achieved by roughly 50% of ID and SD patients (51% and 53%, respectively) and, more seriously, zero in IC patients. Too many operative procedures requiring perfect collaboration of multiple medical teams and difficult exposure of whole IVCTT body are the main reasons for the compromise of R0 resection, especially in IC patients. Furthermore, the more complex the surgery, the higher the perioperative mortalities (2.7%, 6.7%, and 16.7% in ID, SD, and IC patients, respectively). In the long term, the MST for the remaining 60 patients was 18 months, and the 1-, 2-, 3-, and 5-year DSS rates were 76.5%, 41.2%, 26.8%, and 17.1%, respectively. However, when analyzed in subgroups, only 3 (25%) patients in IC survived the first postoperative year while all died within 2 years. For SD patients, DSS was dramatically improved as 10 (66.7%) patients survived the first postoperative year, yet only 1 (6.7%) patient survived 3 years. Best survival benefit was derived from ID patients, where nearly all the patients survived the first postoperative year (91.9%), and achieved 2- and 3-year DSS of 54.1% and 27.0%, respectively. The oncological prognoses of ID patients were comparable to those of previous reports [4, 5, 19], as well as patients with HVTT or PVTT who received surgery [9, 19, 27]. Therefore, curative-intent surgery should take extent of tumor thrombus into consideration among HCC patients with IVCTT, not just conditions of resectable primary tumor and well-preserved physical performance status and liver function [4, 5, 21]. In this study, we propose in the first time that curative-intent surgery for HCC patient with ID, or even SD IVCTT patients, should be considered as one of the preferential treatments, while it might be a futile therapy for IC patients.

Next, we are trying to recognize other risk factors in HCC patients with IVCTT under univariable and multivariable Cox analyses. Unfortunately, we only obtained other four independent risk factors, including ECOG performance status, serum AFP level more than 400 *μ*g/L, tumor size larger than 10 cm, and PVTT status which adversely influenced oncological prognosis. As a matter of fact, plenty of risk factors in previous studies impacting HCC survival were not identified in our study. The possible reasons diversely exit, including overwhelming HBV-related HCC population in China, well-preserved performance status and liver function, relative mild-to-moderate liver cirrhosis in our HCC cohort (data not shown), and effects of other risk factors offset by the extremely advanced disease of HCC and IVCTT [28]. We then constructed a prognostic nomogram for long-term survival after curative-intent surgery for HCC with IVCTT based on independent risk factors identified above. Nomograms can further evaluate multiple risk factors and predict the probability of survival for individual patients [29, 30]. It is important to provide accurate prognostic information to patients, so they can make informed decisions, knowing the chance of achieving a cure and the odds of long-term survival. The nomogram performed well in predicting survival, and the prediction was supported by the C-index (0.812) and the calibration curve. More importantly, we also constructed a dynamic web-based nomogram model, which will be useful and convenient in assisting clinical management and providing visualization of outcome predictions for individual patients at any time and location under the available network.

The present study had several limitations. First, analysis of tumor recurrence was not incorporated into our study. Complete resection of primary tumor and IVCTT was achieved in less than half of the total patients (27/64). It is disputed that patient with IVCTT can never reach curability since tumor thrombus seeds have already disseminated into systemic circulation. It makes no sense to analyze tumor recurrence rate in HCC patient with IVCTT who received surgery simply [31]. The second limitation is inherent to retrospective studies, which are subject to selection bias. In the present study, we observed multiple variations of synchronous resection of primary HCC and IVCTT, which is technically demanding and fraught with risk. For example, patients were heterogeneous in demographic, clinical, and tumor-related characteristics, different surgical techniques were employed for parenchymal dissection or IVCTT removal, and there were various methods of anesthesia management. The third limitation was that our nomogram was developed based on relatively few variables and therefore may represent an imperfect model. Since there were extremely advanced stage HCC patients with IVCTT in our group, many independent risk factors that were proven to affect HCC recurrence and survival in previous reports were not statistically significant in this population. Future studies will be needed to increase the sample size and improve the robustness of the prediction model. Finally, HCC with IVCTT is rare; although we performed rigorous validation of the nomogram using bootstrapped calibration and bias-corrected estimates, future studies will need internal and external validation.

In conclusion, we have constructed an interactive survival prediction nomogram, based on a novel IVCTT classification system, incorporating several independent risk factors, which can make individualized estimates of the survival benefit of curative-intent surgery for HCC patients with IVCTT. This proposed model can assist clinicians and patients in quantifying the potential benefit of curative treatment. To generalize the use of this nomogram, validation with data from other institutions is required.

## Figures and Tables

**Table 1:** Comparison of basic characteristics and clinical variables in three groups.

Characteristics	Type ID (*n* = 37)	Type SD (*n* = 15)	Type IC (*n* = 12)	*P* values (ID vs. SD)	*P* values (SD vs. IC)
Age (years)^*∗*^	53 (35–72)	47 (35–64)	57 (31–78)	0.16	0.26
Gender (male/female)	33 : 4 (89/11)	12 : 3 (80/20)	10 : 2 (83/17)	0.38	0.78
ECOG PS (0/1)	28 : 9 (76/24)	6 : 9 (40/60)	8 : 4 (67/33)	0.01	0.32
Child–Pugh class (A/B)	35/2 (95/5)	13/2 (87/13)	8/4 (67/33)	0.33	0.43
Albumin (g/l)^*∗*^	40.3 (35.0–47.8)	39.7 (33.0–48.5)	38.7 (29.4–47.2)	0.87	0.43
TBil (*μ*mol/l)^*∗*^	14.3 (5.9–43.0)	17.1 (6.7–42.5)	16.4 (8.7–44.7)	0.48	0.77
PT (s)	12.7 (10.3–15.1)	13.4 (11.1–16.7)	12.8 (12.0–13.8)	0.07	0.07
ALT (u/l)^*∗*^	41 (15–118)	37 (21–73)	18 (13–108)	0.37	0.40
Plt (10^∧^^9^/L)^*∗*^	162.5 (56–378)	252 (55–371)	170 (67–373)	0.08	0.20
Perioperative AFP level (<400/≥400 *μ*g/l)	5/32 (14/86)	2/13 (13/87)	2/10 (17/83)	0.99	0.76
Tumor size (cm)^*∗*^	9.8 (5.0–21.0)	11.0 (7.0–15.0)	12.8 (5.0–15.0)	0.25	0.09
<10/≥10 cm	21/16 (57/43)	7/8 (47/53)	1/11 (8/92)	0.51	0.08
Tumor number (solitary/multiple)	25/12 (68/32)	11/4 (73/27)	9/3 (75/25)	0.68	0.73
PVTT (present/absent)	17/20 (46/54)	11/4 (73/27)	7/5 (58/42)	0.07	0.68
Tumor differentiation (well/poor)	16/21 (43/57)	13/2 (12/87)	5/7 (42/58)	0.004	0.04
Radicality (R0/R1-2)	19/18 (51/49)	8/7 (53/47)	0/12 (0/100)	0.90	—

^*∗*^Median (range); values in parentheses are percentages unless indicated otherwise. ID, inferior diaphragmic type; SD, superior diaphragmic type; IC, intracardiac type.

**Table 2:** Comparison of perioperative and long-term oncological outcomes in three groups.

Characteristics	Type ID (*n* = 37)	Type SD (*n* = 15)	Type IC (*n* = 12)	*P* values (ID vs. SD)	*P* values (SD vs. IC)
Operative time (min)	260 (168–515)	307.5 (190–434)	419 (245–460)	0.039	0.004
Blood loss (ml)	950 (400–4000)	1350 (700–8000)	1250 (400–5000)	0.55	0.86
pLOS (d)	12 (8–34)	11 (9–27)	13 (9–26)	0.29	0.14
Morbidity					
CD grade I/II	29 (80.6)	11 (78.6)	10 (100.0)	0.98	0.66
CD grade III	3 (8.3)	1 (7.1)	0 (0.0)	0.69	0.56
CD grade IV	1 (2.7)	2 (14.3)	0 (0.0)	0.40	0.49
90-day mortality	1 (2.7)	1 (6.7)	2 (16.7)	0.90	0.57
Died during follow-up	25 (69.4)	12 (85.7)	8 (80.0)	0.30	0.56
DSS (months)^*∗*^	29 (10–61)	14 (8–40)	8 (4–22)	<0.001	<0.001
DSS rate (%)					
1 year	94.4	27.8	8.3		
2 years	55.6	21.4	0.0		
3 years	71.4	7.1	0.0		
5 years	30.0	0.0	0.0		

^*∗*^Median (range); values in parentheses are percentages unless indicated otherwise. ID, inferior diaphragmic type; SD, superior diaphragmic type; IC, intracardiac type; CD, Clavien–Dindo classification; pLOS, postoperational length of stays in hospital; DSS, disease-specific survival.

**Table 3:** Cox proportional hazards regression model showing the association of characteristics with survival.

Characteristics	Univariate model			Multivariate model		
	*P*	HR	CI95	*P*	HR	CI95
Age (years)	0.58	0.99	0.97–1.02			
Gender (M/F)	0.45	1.44	0.56–3.67			
AFP (*μ*g/ml)						
<400			1.00 (reference)		1.00 (reference)	
≥400	0.023	3.89	1.19–9.69	0.046	3.16	1.02–9.81
ALT (*μ*/l)	0.17	0.99	0.97–1.07			
ALB (g/L)	0.43	1.03	0.96–1.11			
PT (s)	0.28	1.13	0.91–1.40			
Plt (×10…9/l)	0.56	1.00	0.99–1.00			
Tumor size (cm)						
<10		1.00 (reference)			1.00 (reference)	
≥10	<0.001	3.72	1.88–7.37	0.043	2.20	1.02–4.74
Tumor number (solitary/multiple)	0.29	1.40	0.75–2.61			
Differentiation	0.37	0.76	0.42–1.38			
Child–Pugh grade (A/B)	0.009	3.26	1.35–7.88	0.63	1.44	0.33–6.26
TBil (*μ*mol/l)	0.047	1.03	1–1.05	0.17	1.03	0.99–1.07
ECOG performance status (0/1)	<0.001	3.51	1.82–6.77	0.004	2.72	1.37–5.40
PVTT (positive/negative)	0.009	2.27	1.23–4.17	0.002	3.13	1.52–6.41
IVCTT classification						
ID		1.00 (reference)			1.00 (reference)	
SD	0.015	2.39	1.18–2.85	0.25	1.60	0.71–3.60
IC	<0.001	6.85	4.84–16.49	0.001	5.76	2.06–16.07

## Data Availability

The data used to support the findings of this study are available from the corresponding author upon request.

## Supplementary Materials

Supplementary Materials Figure S1: diagnosis algorithm of the HCC patient combined with IVCTT. A: venous phase of magnetic resonance imaging (MRI) displayed huge single lesion (13 cm in diameter, arrowhead) located at the right lobe of the liver, with filling-defect shadow (arrow) at the IVC area of the same section. B: resected sample displayed en bloc resection of the tumor and thrombus (arrow). C: gross sectional specimen. D: microscopic pathology of the HCC and IVCTT (magnification 1000 *μ*m).
