# Supplementary material for: Nomogram for Predicting Long-Term Survival after Synchronous Resection for Hepatocellular Carcinoma and Inferior Vena Cava Tumor Thrombosis: A Multicenter Retrospective Study
Source: J Oncol. 2020 Apr 8;2020:3264079. doi: 10.1155/2020/3264079 (PMC7168703; doi:10.1155/2020/3264079)
Supplement: Supplementary Materials — Figure S1: diagnosis algorithm of the HCC patient combined with IVCTT. A: venous phase of magnetic resonance imaging (MRI) displayed huge single lesion (13 cm in diameter, arrowhead) located at the right lobe of the liver, with filling-defect shadow (arrow) at the IVC area of the same section. B: resected sample displayed en bloc resection of the tumor and thrombus (arrow). C: gross sectional specimen. D: microscopic pathology of the HCC and IVCTT (magnification 1000 μm). [file 3264079.f1.pdf]

Figure S1

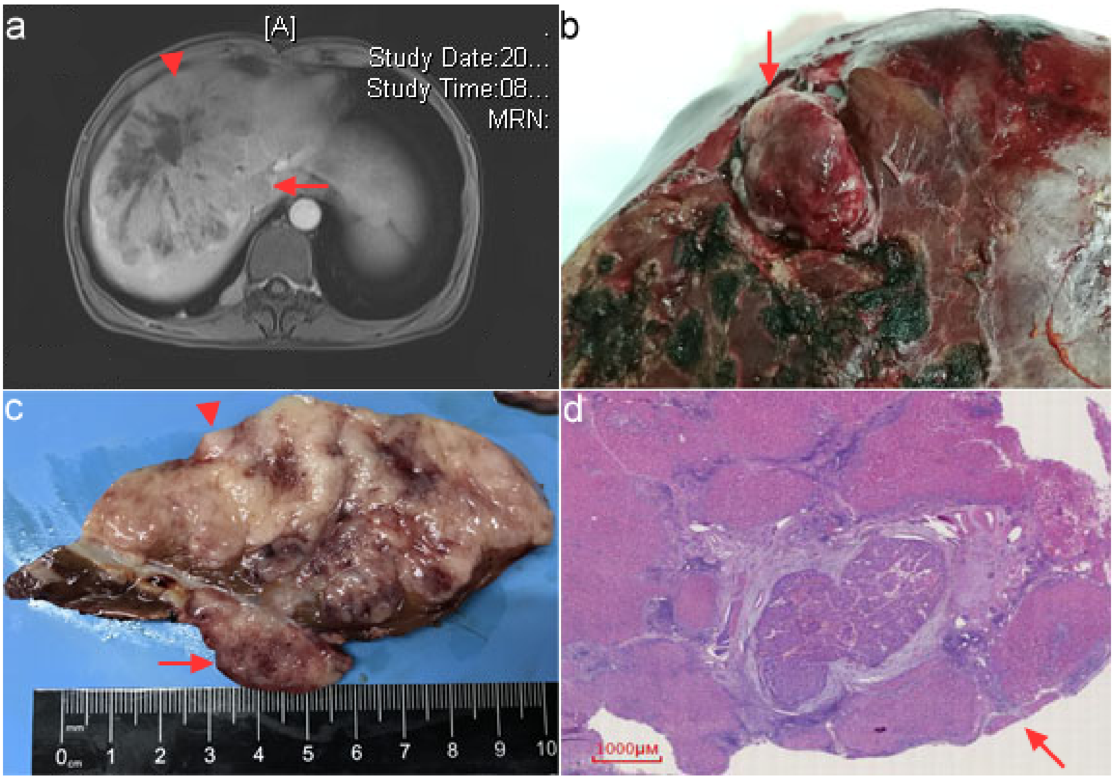

Figure S1

Diagnosis algorithm of HCC patient combined with IVCTT. A Venous phase of magnetic resonance imaging (MRI) displayed huge single lesion (13 cm in diameter, arrowhead) located at right lobe of the liver, with filling-defect shadow (arrow) at IVC area of the same section, B resected sample displayed *en bloc* resection of the tumor and thrombus (arrow), C Gross sectional specimen, D Microscopic pathology of the HCC and IVCTT (magnification 1000 μm).
